# Built Environment Features and Cardiometabolic Mortality and Morbidity in Remote Indigenous Communities in the Northern Territory, Australia

**DOI:** 10.3390/ijerph19159435

**Published:** 2022-08-01

**Authors:** Amal Chakraborty, Margaret Cargo, Victor Maduabuchi Oguoma, Neil T. Coffee, Alwin Chong, Mark Daniel

**Affiliations:** 1 University Centre for Rural Health, The University of Sydney, Lismore, NSW 2480, Australia; 2 Health Research Institute, Faculty of Health, University of Canberra, Bruce, ACT 2601, Australia; margaret.cargo@canberra.edu.au (M.C.); n.coffee@deakin.edu.au (N.T.C.); dr.mark.daniel@gmail.com (M.D.); 3 Poche Centre for Indigenous Health, Faculty of Health and Behavioural Sciences, The University of Queensland, St Lucia, QLD 4067, Australia; v.oguoma@uq.edu.au; 4 Deakin Rural Health, Rural Health Multidisciplinary Training (RHMT) Program, School of Medicine, Deakin University, Warrnambool, VIC 3280, Australia; 5 Australian Centre for Housing Research, The University of Adelaide, Adelaide, SA 5005, Australia; 6 Arney Chong Consulting, Adelaide, SA 5081, Australia; alwin.chong@arneychongconsulting.com

**Keywords:** built environment, cardiovascular disease, remote community, health care, Aboriginal and Torres Strait Islanders, epidemiology

## Abstract

Indigenous Australians experience poorer health than non-Indigenous Australians, with cardiometabolic diseases (CMD) being the leading causes of morbidity and mortality. Built environmental (BE) features are known to shape cardiometabolic health in urban contexts, yet little research has assessed such relationships for remote-dwelling Indigenous Australians. This study assessed associations between BE features and CMD-related morbidity and mortality in a large sample of remote Indigenous Australian communities in the Northern Territory (NT). CMD-related morbidity and mortality data were extracted from NT government health databases for 120 remote Indigenous Australian communities for the period 1 January 2010 to 31 December 2015. BE features were extracted from Serviced Land Availability Programme (SLAP) maps. Associations were estimated using negative binomial regression analysis. Univariable analysis revealed *protective effects* on all-cause mortality for the BE features of Education, Health, Disused Buildings, and Oval, and on CMD-related emergency department admissions for the BE feature Accommodation. Incidence rate ratios (IRR’s) were greater, however, for the BE features Infrastructure Transport and Infrastructure Shelter. Geographic Isolation was associated with elevated mortality-related IRR’s. Multivariable regression did not yield consistent associations between BE features and CMD outcomes, other than negative relationships for Indigenous Location-level median age and Geographic Isolation. This study indicates that relationships between BE features and health outcomes in urban populations do not extend to remote Indigenous Australian communities. This may reflect an overwhelming impact of broader social inequity, limited correspondence of BE measures with remote-dwelling Indigenous contexts, or a ‘tipping point’ of collective BE influences affecting health more than singular BE features.

## 1. Introduction

Despite policies aimed at reducing health inequity [1], the health of Aboriginal and Torres Strait Islander peoples (hereafter respectfully referred to as Indigenous Australians) remains poorer than that of non-Indigenous Australians [2]. Non-communicable chronic diseases, particularly cardiometabolic disease (CMD), which includes cardiovascular disease (CVD), diabetes, and chronic kidney disease (CKD), are the largest contributors to gaps in life expectancy, mortality and disease burden between Indigenous and non-Indigenous Australians. CMD accounts for over two-thirds (70%) of the health gap between Indigenous and non-Indigenous Australians [3,4]. Some 38% of Indigenous Australian adults experience two or more of the cardiometabolic conditions of CVD, diabetes or CKD, compared to 26% of non-Indigenous adults [5]. The proportion of hospitalizations among people with all three cardiometabolic conditions is also higher in the Indigenous (18%) compared to non-Indigenous population (7%) [5]. In addition, the prevalence of chronic diseases and resultant hospitalisation rates are higher for Indigenous people living in very remote, remote, and outer regional areas compared to Indigenous people living in cities and inner regional Australia [6,7,8,9,10,11].

The basis of Indigenous-non-Indigenous health disparity in Australia is similar to that elsewhere, notably North America and Northern Europe [12,13]. The health conditions experienced by many Indigenous Australians are shaped by the broader social and cultural determinants in which they live [14,15]. This includes, but is not limited to, employment, income, housing, community, and structural factors; and other resources that promote health and wellbeing such as relationships to culture and spirituality, connection to country, family, and community-connections that have survived despite colonization attempts to disrupt the social and cultural fabric of Indigenous Australians [15,16]. For Indigenous Australians, good health is not merely a matter of the provision of access to clinicians, health services and medications, or the absence of disease, but holistic wellbeing of the whole community including determining all aspects of life, such as control over the physical environment, dignity, self-esteem and justice [17]. Dependency on government reinforces the marginal place of Indigenous people vis-à-vis mainstream society, evidenced by rising absolute and relative cardiovascular mortality inequalities that for Indigenous people track with neoliberal policy reforms [18].

Existing research tends to attribute the development of chronic disease in Indigenous Australians to individual-level behavioural ‘risk factors’ [19,20,21,22,23]. However, these studies generally overlook the quality of the places where people live and the opportunities these provide for making healthful choices shaping people’s health behaviour, and thus enabling development (or not) of risk factors for chronic disease in Indigenous communities [24]. In other words, individual-level behavioural ‘risk factors’ reflect underlying collective exposure to ‘risk conditions’—that is, unfavourable built, social, cultural and political environmental contexts [24,25] (per the above). Maintaining healthful environmental living conditions is thus pivotal to addressing immediate to longer-term risks for high prevalence chronic diseases. The World Health Organisation (WHO) now calls for creating and maintaining healthful environments as a priority for primary prevention of diseases [26]. This call is supported by the Indigenous leadership who have long been advocating for improvements in living conditions in remote Indigenous communities in Australia [27,28,29].

The built environment (BE) (i.e., local area infrastructure, including housing, retail food sources and recreational areas) [25] is now well established as linked to cardiovascular (CVD) risk and CVD outcomes in urbanised populations [30]. The BE is important, as for exposed individuals its features can either exacerbate or counteract an underlying social vulnerability to risk or disease. The environment conditions the expression of individual risk, and thus the collective frequency and distribution of morbidity and mortality for defined communities. This perspective on broader environmental influences enables viewing the burden of CMD affecting Indigenous peoples in terms of larger forces dictating the nature and quality of health-relevant community BE features [24].

Despite calls for evaluating the relationship between BE features and CMD risk in remote Indigenous Australian populations [25], such work is scarce compared to the literature investigating these relationships across urban contexts [11,31,32]. Literature addressing the BE in remote Indigenous Australian communities has focused on deficits in housing [33] and housing-related health hardware (e.g., sanitation and hygiene capabilities) [34,35,36]. Some recent work has investigated the influence of the non-housing related BE on disease outcomes in remote Indigenous communities [37,38,39,40] in Australia, offering promising directions for population health improvement. Such literature is, however, limited, and a knowledge gap remains on the impact of non-housing related BE features on cardiometabolic health, particularly for remote Indigenous communities in the Northern Territory (NT) of Australia. The primary contribution of this study was to evaluate the impact on cardiometabolic outcomes of broader, non-housing related environmental factors relevant to chronic disease. A better understanding of these relationships has the potential to guide contextual intervention to reduce the health inequities experienced by remote Indigenous Australian populations. 

The aim of this study was to estimate associations between BE features and CMD-related morbidity and mortality rates in remote, predominantly Indigenous communities in the NT of Australia. 

## 2. Materials and Methods

### 2.1. Study Design and Ethics

This study was part of the Environments and Remote Indigenous Cardiometabolic Health (EnRICH) project, a cross-sectional epidemiological study conducted between 2013 and mid-2018 using aggregated geographic and community-level health outcomes data. Ethical approval for this study was obtained from the Human Research Ethics Committee (HREC) of the University of South Australia (#31875, #33207), Central Australian HREC (#13-182), HREC of the Northern Territory Department of Health and the Menzies School of Health Research (#2013-2083) and the University of Canberra HREC (blanket cross-institutional approval, 20 June 2017). 

### 2.2. Setting

Eight hundred and thirty-three Indigenous Australian communities in the NT were identified through the Australian Government Indigenous Programs and Policy Location (AGIL) 2013 dataset. Designated remote communities [41] meeting the following criteria were included: population size ≥50 persons (loss *n* = 693 communities) and ≥70% of community residents Indigenous Australian (loss *n* = 17). 

The resultant 123 eligible communities were matched to 104 Indigenous locations (ILOCs, the smallest resolution at which census data for the Indigenous Australian population are available from the Australian Bureau of Statistics (ABS) [41]) enabling sociodemographic data (population size, mean age) for each ILOC to be extracted from the ABS 2011 Census of Population and Housing [42]. Of the 104 ILOCs, 13 contained more than one AGIL-defined community accounting for an “extra” 19 AGIL-defined communities. The unit of observation and analysis was the ILOC. Where multiple communities were present within an ILOC, community-level outcome and BE exposure data were aggregated to create ILOC-level data. 

### 2.3. Outcome Data

Data for five outcome measures were aggregated for the period 1 January 2010 to 31 December 2015: (1) counts of CMD-related visits to NT Primary Healthcare centres, from the Primary Healthcare Collection of the NT Department of Health; (2) counts of CMD-related inpatient admissions, from the Inpatient Activity of the NT Department of Health; (3) counts of CMD-related admissions recorded by emergency departments, from the Emergency Department Data Collection of the NT Department of Health; (4) counts of deaths deemed to be caused by CMD, from the NT-wide mortality dataset of the Births, Deaths, and Marriages office (NT Department of the Attorney General and Justice); and (5) counts of all deaths, from the NT-wide mortality dataset of the Births, Deaths, and Marriages office (NT Department of the Attorney General and Justice). 

Full details of ICD-10 (for CMD-related mortality and all-cause mortality) and ICPC-2 codes (for CMD-related Inpatient Admissions, CMD-related Emergency Department related admissions and CMD-related visits to NT Primary Healthcare Centres) defining each outcome measure are presented in supplementary materials (see Supplementary Data, file Table S1). Counts were assigned to communities using the ‘usual community of residence’ record field. CMD was classified as including hypertension, stroke, CVD, and type-2 diabetes mellitus.

The highest outcome data coverage was for mortality (all-cause and CMD-related, *n* = 95 ILOCs), followed by CMD-related inpatient admission (*n* = 89 ILOCs), CMD-related emergency department admissions (*n* = 77 ILOCs), and CMD-related primary healthcare visits (*n* = 69 ILOCs). Lower coverage for primary healthcare visits exists as the data source (NT Department of Health) does not capture data for non-governmental (i.e., for Aboriginal community controlled) primary healthcare centres that operate in the NT [37]. Outcomes were analysed relative to ILOC-specific population denominators [42].

### 2.4. Exposures Data

Counts of buildings and other infrastructure (hereafter, ‘feature(s)’) were extracted from Serviced Land Availability Programme (SLAP) maps maintained by the NT Department of Lands, Planning, and the Environment. These maps are compiled from data held by, inter alia, NT Department of Lands, Planning and the Environment, NT Department of Housing, Local Government and Regional Services, Power and Water Corporation, and the Aboriginal Areas Protection Authority, and show individual BE features at small scales (typically 1:2000 to 1:2500). Map dates depicted BE features present before and during the outcome measure sample period. Each feature was assigned to a category based on its function, purpose, or status [37].

Non-exhaustive characterizations of the predominant building types in each category are as follows. ‘Accommodation’ captures collective accommodation infrastructure, typically for transitory workers, e.g., ‘Single men’s quarters’, ‘hostels’ and ‘contractor’s accommodation’. This category does not capture private homes. ‘Aged Care’ captures aged care facilities. ‘Child Care’ captures childcare centres and creches. ‘Community’ captures women’s centres, art centres, community halls and libraries. ‘Disused buildings’ primarily consists of ruins. ‘Education’ is dominated by Primary and Secondary schools, but also includes Technical and Further Education, vocational and adult education centres. ‘Services’ primarily consists of law enforcement-related facilities such as police stations, courts and jails. ‘Health’ covers health centres and clinics. ‘Industry’ captures workshops, fuel depots, and abattoirs and meatworks structures. ‘Infrastructure Sewage’ comprises sewage pumping stations exclusively. ‘Infrastructure Shelter’ was almost exclusively comprised of shade shelters. ‘Infrastructure Transport’ was exclusively comprised of bus shelters. ‘Religion’ was almost exclusively comprised of churches. ‘Retail’ comprised stores, shops, and (rarely) supermarkets and service stations. ‘Sport and recreation’ comprised sport and recreation halls and clubs, in addition to basketball courts. The category ‘Arena’ comprises sports and physical recreation venues or grounds with associated facilities (e.g., spectator seating, changerooms), whereas ‘Ovals’ comprise outdoor sports fields without associated facilities. ‘Storage’ consists of storage sheds and areas as well as cold stores. ‘Unfinished Buildings’ comprised incomplete buildings exclusively. 

A geographic isolation measure was determined for each community, defined as the presence or absence of another AGIL community within 300 km along the mainland road network). Geographic distance between remote communities was measured at multiple distances, starting with nearby communities and with progressively increasing road network radii (10 km, 50 km, 100 km, 200 km, 300 km, and 500 km). As the 300 km distance was most strongly associated with outcomes it was used as the geographic isolation measure.

### 2.5. Statistical Analysis

Given non-parametric distributions, exposures were either median split into a dichotomous variable or re-categorised as the presence or absence of a BE feature. Case-wise exclusion of ILOCs lacking exposure data (*n* = 3) reduced the analytic sample to *n* = 101 ILOCs. A negative binomial (NB) regression model with the population of the ILOC as an exposure variable (offset) was used to estimate the univariable association between BE features and outcomes. 

For multivariable modelling, BE categories were grouped into six domains: (a) ‘Shelter’, comprising Infrastructure Transport and Shelter features; (b) ‘Community’, comprising Accommodation, Aged Care, Childcare and Community features; (c) ‘Services’, comprising Services, Education and Health features; (d) ‘Commercial’, comprising Industry and Retail features; (e) ‘Sports and Recreation’, comprising Arenas, Ovals, and Sports and recreation features; and (f) ‘Disused’, consisting of the ‘Disused’ category of buildings only. The commercial category was not separated from the industrial classification as in the remote Indigenous context ‘industry’ and ‘commercial’ are blended.

These six BE domains together with the community geographic isolation measure resulted in seven separate models. Aggregation of features to domains was based on univariable regression outcomes of *p* < 0.20 and/or similarity of purpose of features. The categories ‘Infrastructure Sewage’, ‘Religion’, ‘Unfinished’, and ‘Storage’ features were omitted from the multivariable regression model due to their low frequency and unclear *a priori* direction of impact on physical health (e.g., churches). For instance, sewage pumping stations were only evident in 3% of communities as this facility is often located away from the community and does not feature in the community focused SLAP map.

In the multivariable model, domains and the geographic isolation measure were further adjusted for ILOC median age, and features were assessed by domain. In both univariable and multivariable analyses, associations between BE features and outcomes are reported as incidence rate ratios (IRR) with a 95% confidence interval (CI). Statistical significance was set at α = 0.05. Analyses were conducted using Stata 16.1 (StataCorp, College Station, TX, USA).

## 3. Results

ILOC-level descriptive data for the population and built environment are presented in Table 1. Median ILOC population size was 220 (25th–75th percentile, 129–426); and the median age was 24 years (25th–75th percentile, 22–26). The majority of ILOCs had another AGIL community within 300 km (88.1%). BE features having limited representation across ILOCs included Accommodation (32.7%), Aged care (10.9%), Childcare (13.9%), Services (31.7%), or Sports and recreation (34.7%). Oval (79.5%), Arena (77.1%), Education (66.3%) and Health (63.4%) features were more frequent. 

In univariable analyses, BE features inversely associated with all-cause mortality included Education (IRR 0.51 (95% CI: 0.32–0.81), *p* = 0.004), Health (0.55 (95% CI: 0.35–0.87), *p* = 0.010), Disused buildings (0.59 (95% CI: 0.38–0.90), *p* = 0.015), Oval (0.60 (95% CI: 0.38–0.95), *p* = 0.028) and, although weak, Community (0.66 (95% CI: 0.43–1.03), *p* = 0.065). All-cause mortality in communities with these BE features ranged from 34–49% lower than those without. CMD-related emergency department admission rates were inversely associated with Accommodation features (0.53 (95% CI: 0.33–0.85), *p* = 0.009). All-cause mortality was positively associated with Infrastructure transport features (2.33 (95% CI: 1.23–4.39), *p* = 0.009), CMD-related inpatient admissions rates were positively associated with Infrastructure shelter features (1.57 (95% CI: 1.03–2.40), *p* = 0.035), and CMD-related emergency department admission rates were positively associated with Childcare features (1.64 (95% CI: 0.91–2.96), *p* = 0.099) (Table 2). 

Similarly, in univariable analyses, Geographic Isolation was strongly positively associated with greater risk of both all-cause (5.34 (95% CI: 1.80–15.90), *p* = 0.003) and CMD-related mortality (6.73 (95% CI: 1.24–36.37), *p* = 0.027). There were no statistically significant associations between BE features and CMD-related mortality or CMD-related primary healthcare visits, although an elevated rate of CMD-related primary health care visits (~50% increase) was observed in the presence of Aged Care features (1.54 (95% CI: 0.78–3.01, *p* = 0.212) (Table 2).

In multivariable analyses, a unit increase in the median age of ILOC residents was associated with a greater risk of morbidity and mortality except for CMD-related inpatient admissions (Table 3). Associations between geographic isolation and disease outcomes were comparable to those identified in the univariable analysis, with mortality-related outcomes retaining positive associations (IRR’s for all-cause mortality (4.68 (95% CI: 1.55–14.16), *p* = 0.006) and CMD-related mortality (6.10 (95% CI: 1.12–33.11), *p* = 0.036)). Positive associations were evident between the presence of an Arena and both all-cause mortality (1.95 (95% CI: 1.21–3.15), *p* = 0.006) and CMD-related primary healthcare visits (2.03 (95% CI: 1.05–3.91), *p* = 0.034). Positive associations were also evident between the presence of an Oval and CMD-related emergency department admissions (2.05 (95% CI: 1.11–3.79), *p* = 0.021), the presence of Infrastructure Transport and all-cause mortality (2.14 (95% CI: 1.11–4.13), *p* = 0.023), and the presence of Infrastructure Shelter and CMD-related inpatient admissions (1.60 (95% CI: 1.05–2.44), *p* = 0.030). Negative or protective relationships were observed between the presence of Accommodation and CMD-related emergency department admissions (0.58 (95% CI: 0.34–0.99), *p* = 0.044), and the presence of Disused buildings and all-cause mortality (0.65 (95% CI: 0.42–1.00), *p* = 0.048) (Table 3).

## 4. Discussion

This study is one of very few to address a lack of attention to relationships between BE features and CMD-related mortality and morbidity in remote Indigenous communities in Australia. It is exceptional in scope, including 120 communities within 101 ILOCs. No consistent or strongly defensible *patterns* of associations were observed, except for predictable associations between age, geographic isolation and mortality outcomes, and between age and two of three morbidity outcomes. Notwithstanding some more-or-less sporadic, non-patterned associations of other BE features with a limited number of the five outcomes assessed, the primary finding of this study is that relationships between BE features and CMD-related outcomes, well-established for urban Australian communities, appear not to extend to remote Indigenous Australian communities.

We propose three interpretations for the absence among remote Indigenous communities of patterns of associations between BE features and CMD-related morbidity and mortality that are now well-established for urban populations in Australia, and throughout the world. First, CMD in remote Indigenous Australian communities may be primarily shaped by broad and highly substantial social disparity vis-à-vis Australian society [43], more than by BE features that vary between communities. While functional built environments are logically important even if not implicated here, the limited extent of patterned associations in this analysis could suggest that reducing cardiometabolic disparities in remote Indigenous Australian communities might be best promoted through creative, supportive environments and the strengthening of community action [44], and by recognising the important role of the social environment in shaping chronic disease outcomes through reciprocal relationships with behaviour, including participation in community and cultural activities [45], and cognition [46,47]. 

Second, measures of BE infrastructure used in this study may not adequately correspond to the remote-dwelling Indigenous context. A well-developed taxonomy of the BE for the remote Indigenous Australian context is lacking, despite policies [48,49] that target improvements in the BE. Most studies approach the broader BE–health relationship (which includes a focus on CMD) from an urban, Western perspective [30], which is neither necessarily applicable to, nor optimised for, remote Indigenous Australian communities [50]. Participatory processes could be used to engage Indigenous Australian communities in the design of culturally relevant and meaningful BE measures [51]. 

Third, the impact of the BE on cardiometabolic morbidity and mortality could be through a ‘tipping point’ of collective BE influences, more than singular, “independent” features. This could arise in the way the BE provides an adequacy (or not) of overall resources and opportunities to enable health. The lack of a consistent pattern of association for most BE features in this study reflects their insufficient strength of association as “independent” resources and opportunities relating to CMD. It may be that the BE shapes CMD in this context only through a ‘tipping point’ of *collective* BE influences (e.g., Le Gal et al. [37]). Further research is required, however, before conceding this speculation, given that the literature on non-housing-related BE features in the remote, Indigenous context is sparse.

Our observations should not be interpreted as a support for restricting investment in the BE of remote Indigenous Australian communities. Indeed, we would contend the opposite: Our results, together with other approaches by which the overall BE is scored overall, rather than unique effects estimated [37], supports efforts to broaden the range of (healthful) BE features to which a given community has access. It may be that the BE requires an accumulation of different features to exert a healthful community-level effect: a broader, rather than lesser, range of BE features may be indicated to elicit health benefits from the BE. Unfortunately, this conceptualisation is not congruent with government approaches which focus on the creation of a ‘hub and spoke’ model of resourcing, with predictably inevitable reductions in services at the ‘spoke’ communities (i.e., the remote communities that are the subject of this study), and an expectation of temporary or permanent migration toward the ‘hub’ towns for access to services [52]. Service loss in smaller remote communities is reported in neighbouring Western Australia [53]. We would speculate that the narrowing of the range of BE features in any given community that occurs consequent to such service loss is unlikely to enhance the potential for the BE to exert positive effects on Indigenous Australian health. We excluded very small (n < 50 persons) communities from our analysis and thus our conclusions pertain to a range of community sizes above this minimum.

The findings from this study provide a case for developing further research to understand the health relevance of heterogeneity in built environmental living conditions in remote Australian Indigenous communities [54,55,56]. While it is important to examine Indigenous peoples’ health behaviour and its relationship to disease, it is equally if not more important to understand the contexts that produce such behaviour. These constitute the environmental living conditions, opportunities and resources available in the places where Indigenous people grow, live, and work [24,57,58]. Examining heterogeneity in built environmental living conditions within and between small-area or wider community locations will provide a necessary understanding of how features of community built environments relate to CMD in remote contexts to better inform government policy and service delivery [24]. However, there is a limited extent of good quality publicly available community-level built environmental data which can be utilised in large scale inferential analyses to evaluate relationships with disease prevalence and outcomes. This study used NT Government SLAP maps, as noted. Whilst useful, this data source arguably could be supplemented with more expansive BE feature representations that are scientifically valid in terms of construct, content and criterion validity, as well as being culturally relevant at the same time. Limitations in data availability and quality are consistent with the call from Indigenous Australian leaders who have highlighted a strong need for community-level data to facilitate local decision making that affects local services [59]. Our research demonstrates that the compiling of such community-level built environmental data is not only possible but essential for evaluating relationships with the prevalence of diseases [40,60,61]. Providing such data for analysis is important to enable decision-makers to make better-informed, evidence-based decisions to guide health and social actions to benefit remote Indigenous communities in Australia [62]. 

This study is subject to several caveats. The mobility of some Indigenous Australian populations [63] could compromise the validity of the field code “usual” community of residence. Such imprecision will have biased our results towards the null. It is also likely that limited heterogeneity in BE measures across ILOCs reflects the need to develop more theoretically and conceptually appropriate measures [50], ideally through engagement with community residents [51]. This need is supported by our observation of a small number of non-patterned, counterintuitive associations, e.g., the presence of an Arena was positively associated with all-cause mortality and CMD-related primary healthcare visits. Such findings could reflect either measurement limitations or, if reproducible, a need to clarify the basis of this relationship (e.g., social norms shaping CMD may be expressed through collective activities at Arenas). While there is a firm conceptual and theoretical basis underpinning a relationship between BE features and CMD in Indigenous Australian communities [25], measurement of the BE remains challenging given the need to develop scientifically valid and culturally relevant measures of the BE [50,51,64]. As the study was sufficiently powered to enable the consistent identification of the relatively small effects of median age, we interpret it is unlikely (as small effects could be identified for age) that the study was underpowered to detect the effects of individual BE features. 

## 5. Conclusions

This study evaluating relationships between community-level BE features and CMD outcomes observed defensible *patterns* of associations between age, geographic isolation and all-cause and CMD-specific mortality, and between median age and two of the three morbidity outcomes investigated. Univariate results indicated that the BE features of Education, Health, Oval and Disused buildings were protective against all-cause mortality, with Accommodation features protective for CMD-related emergency department admissions. Infrastructure transport and Infrastructure shelter features were risk factors for all-cause mortality and CMD-related inpatient admissions, respectively. Geographic isolation, in both univariate and multivariable analyses, was a strong risk factor for all-cause and CMD-related mortality. Multivariable analyses further indicated that a unit increase in the median age of ILOCs was associated with greater morbidity and mortality for four of five outcome measures (the exception was CMD-related inpatient admissions). The presence of an Arena was positively associated with both all-cause mortality and CMD-related primary healthcare visits. Multivariable analysis did not implicate other BE features. Apparently random associations were observed, limited to no more than one of the five outcome measures considered, for other BE features including Infrastructure shelter, Infrastructure transport, Accommodation, Oval, and Disused buildings. The primary finding of this study is that relationships between BE features and CMD-related outcomes, well-established for urban Australian communities, appear not to extend to remote Indigenous Australian communities. This may reflect an overwhelming impact of broader social inequity, the limited correspondence of BE measures with remote-dwelling Indigenous contexts, or a ‘tipping point’ of collective BE influences affecting health more than singular BE features. The implications of these results include a need for BE measurement development to achieve multi-dimensional indicators that are scientifically valid and meaningful to remote Indigenous communities [50,51]. Such indicators should be sufficiently sensitive in accounting for the possibility that the contribution of BE features to CMD in remote Indigenous Australian communities may be secondary to a broader impact of substantial social disparity vis-à-vis Australian society. Functional built environments are logically important even if not implicated here. Hence, the limited extent of patterned associations in this study could suggest against the mounting of narrowly conceived interventions. Rather, proactive efforts to reduce cardiometabolic disparities in remote Indigenous Australian communities might best take the form of initiatives promoting creative, supportive environments and the strengthening of community action, recognising the important role of the social environment in shaping chronic disease outcomes through reciprocal relationships with behaviour, including participation in community and cultural activities.

## Figures and Tables

**Table 1:** ILOC-level descriptive data.

Characteristic of ILOC (*n* = 101)	Percentage of ILOC’s with Feature Type
Infrastructure transport	11.9%
Infrastructure shelter	51.5%
Infrastructure sewage	3.0%
Accommodation	32.7%
Community	56.4%
Age care	10.9%
Childcare	13.9%
Education	66.3%
Health	63.4%
Services	31.7%
Industry	55.4%
Retail	51.5%
Religion	43.6%
Sports and recreation	34.7%
Arena	77.1%
Oval	79.5%
Storage	16.8%
Unfinished building	59.4%
Disused buildings	52.5%
Geographic Isolation *	88.1%
ILOC remoteness and population characteristics	Median (Q1–Q3)
Accessibility Remoteness Index Australia	13.77 (11.18–14.96)
Age (years)	24 (22–26)
ILOC population size	220 (129–426)

Note: *—mainland communities only; Q1 = 25th percentile Q3 = 75th percentile.

**Table 2:** Univariable regression of built environment features on all-cause mortality and cardiometabolic-related mortality and morbidity.

	All-Cause Mortality (*n* = 95)		CMD-Related Mortality (*n* = 95)		CMD-Related ED Admissions (*n* = 77)		CMD-Related Primary Health Care Visits (*n* = 69)		CMD-Related Inpatient Admissions (*n* = 89)	
	IRR (95% CI)	*p*	IRR (95% CI)	*p*	IRR (95% CI)	*p*	IRR (95% CI)	*p*	IRR (95% CI)	*p*
Infrastructure transport	**2.33 (1.23, 4.39)**	**0.009**	1.95 (0.96, 3.95)	0.064	1.28 (0.65, 2.52)	0.478	0.87 (0.42, 1.83)	0.716	1.35 (0.72, 2.55)	0.354
Infrastructure shelter	1.37 (0.89, 2.12)	0.155	1.22 (0.73, 2.02)	0.447	1.42 (0.89, 2.27)	0.143	0.85 (0.53, 1.38)	0.521	**1.57 (1.03, 2.40)**	**0.035**
Infrastructure sewage	0.72 (0.22, 2.40)	0.597	1.12 (0.30, 4.16)	0.861	1.32 (0.41, 4.25)	0.644	1.38 (0.43, 4.41)	0.583	1.79 (0.57, 5.69)	0.321
Accommodation	0.72 (0.46, 1.13)	0.152	1.00 (0.60, 1.67)	0.988	**0.53 (0.33, 0.85)**	**0.009**	1.02 (0.63, 1.65)	0.935	0.75 (0.48, 1.16)	0.194
Community	0.66 (0.43, 1.03)	0.065	0.84 (0.50, 1.39)	0.494	1.11 (0.67, 1.83)	0.682	1.14 (0.66, 1.96)	0.637	1.40 (0.90, 2.15)	0.133
Age care	0.89 (0.46, 1.71)	0.721	1.01 (0.50, 2.05)	0.971	1.16 (0.60, 2.22)	0.663	1.54 (0.78, 3.01)	0.212	1.11 (0.59, 2.09)	0.752
Childcare	0.84 (0.47, 1.52)	0.568	1.07 (0.56, 2.05)	0.834	1.64 (0.91, 2.96)	0.099	1.12 (0.61, 2.06)	0.710	1.42 (0.80, 2.53)	0.226
Education	**0.51 (0.32, 0.81)**	**0.004**	0.62 (0.36, 1.06)	0.082	0.96 (0.55, 1.67)	0.872	0.76 (0.40, 1.46)	0.415	1.19 (0.74, 1.91)	0.476
Health	**0.55 (0.35, 0.87)**	**0.010**	0.72 (0.42, 1.23)	0.232	0.91 (0.53, 1.57)	0.745	0.89 (0.45, 1.74)	0.727	1.23 (0.78, 1.93)	0.369
Services	0.90 (0.57, 1.41)	0.635	1.09 (0.66, 1.80)	0.748	1.32 (0.82, 2.12)	0.247	1.01 (0.63, 1.63)	0.957	1.30 (0.83, 2.02)	0.249
Industry	0.68 (0.44, 1.06)	0.086	0.86 (0.51, 1.43)	0.556	0.80 (0.49, 1.32)	0.384	1.00 (0.57, 1.73)	0.988	1.01 (0.66, 1.56)	0.955
Retail	0.76 (0.49, 1.16)	0.205	0.94 (0.57, 1.56)	0.809	0.94 (0.58, 1.52)	0.799	1.16 (0.68, 1.98)	0.580	1.17 (0.77, 1.78)	0.472
Religion	0.81 (0.53, 1.24)	0.334	1.07 (0.65, 1.76)	0.786	1.12 (0.71, 1.78)	0.624	0.99 (0.61, 1.61)	0.969	1.22 (0.80, 1.85)	0.360
Sports and recreation	0.71 (0.46, 1.11)	0.132	0.81 (0.49, 1.33)	0.409	0.97 (0.61, 1.53)	0.888	0.80 (0.50, 1.28)	0.353	1.20 (0.78, 1.84)	0.404
Arena	1.40 (0.89, 2.21)	0.145	1.09 (0.64, 1.85)	0.764	0.97 (0.58, 1.61)	0.902	1.26 (0.70, 2.29)	0.439	1.36 (0.88, 2.12)	0.167
Oval	**0.60 (0.38, 0.95)**	**0.028**	0.67 (0.39, 1.15)	0.147	**2.02 (1.16, 3.53)**	**0.013**	0.88 (0.47, 1.66)	0.703	1.43 (0.91, 2.26)	0.124
Storage	0.68 (0.39, 1.18)	0.171	0.81 (0.43, 1.52)	0.515	0.77 (0.43, 1.37)	0.368	1.13 (0.61, 2.12)	0.697	0.92 (0.53, 1.58)	0.758
Unfinished building	0.68 (0.44, 1.05)	0.083	0.95 (0.57, 1.59)	0.847	0.83 (0.52, 1.34)	0.450	0.95 (0.58, 1.58)	0.851	0.96 (0.63, 1.48)	0.866
Disused buildings	**0.59 (0.38, 0.90)**	**0.015**	0.70 (0.43, 1.15)	0.155	1.12 (0.70, 1.79)	0.636	1.02 (0.62, 1.69)	0.923	1.27 (0.83, 1.93)	0.268
Geographic Isolation *	**5.34 (1.80, 15.90)**	**0.003**	**6.73 (1.24, 36.37)**	**0.027**	1.62 (0.38, 6.97)	0.518	0.86 (0.12, 6.24)	0.881	1.76 (0.70, 4.43)	0.227

Note—boldface indicates statistical significance at *p* = 0.05. CMD—Cardiometabolic disease, CI—confidence intervals, ED—emergency department, IRR—Incidence Rate Ratio, *—mainland communities only.

**Table 3:** Multivariable regression of built environment features by domain on all-cause mortality and cardiometabolic-related mortality and morbidity, adjusting for median age of ILOC population.

	All-Cause Mortality (*n* = 95)		CMD-Related Mortality (*n* = 95)		CMD-Related ED Admissions (*n* = 77)		CMD-Related Primary Health Care Visits (*n* = 69)		CMD-Related Inpatient Admissions (*n* = 89)		Models
	IRR (95% CI)	*p*	IRR (95% CI)	*p*	IRR (95% CI)	*p*	IRR (95% CI)	*p*	IRR (95% CI)	*p*	
Shelter (Model 1)											Model 1
Median age	**1.07 (1.02, 1.12)**	**0.003**	**1.10 (1.04, 1.17)**	**0.002**	**1.09 (1.03, 1.16)**	**0.006**	**1.10 (1.02, 1.17)**	**0.008**	1.01 (0.96, 1.07)	0.682	
Infrastructure shelter	1.27 (0.81, 2.00)	0.300	1.38 (0.81, 2.35)	0.241	1.57 (0.98, 2.52)	0.062	0.99 (0.61, 1.60)	0.956	**1.60 (1.05, 2.44)**	**0.030**	
Infrastructure transport	**2.14 (1.11, 4.13)**	**0.023**	1.96 (0.96, 4.01)	0.064	1.37 (0.69, 2.70)	0.367	0.88 (0.42, 1.84)	0.733	1.36 (0.72, 2.56)	0.347	
Community (Model 2)											Model 2
Median age	**1.07 (1.01, 1.12)**	**0.012**	**1.09 (1.02, 1.15)**	**0.007**	1.06 (0.99, 1.13)	0.080	**1.09 (1.02, 1.17)**	**0.014**	0.99 (0.93, 1.05)	0.708	
Accommodation	0.90 (0.55, 1.46)	0.658	1.19 (0.69, 2.06)	0.523	**0.58 (0.34, 0.99)**	**0.044**	1.08 (0.65, 1.79)	0.772	0.65 (0.40, 1.06)	0.087	
Age care	0.93 (0.48, 1.80)	0.819	0.95 (0.47, 1.95)	0.894	1.32 (0.64, 2.73)	0.453	1.32 (0.65, 2.67)	0.439	1.32 (0.66, 2.62)	0.435	
Childcare	0.91 (0.49, 1.68)	0.763	1.10 (0.57, 2.13)	0.768	1.34 (0.73, 2.48)	0.346	0.94 (0.49, 1.80)	0.850	1.26 (0.70, 2.28)	0.443	
Community	0.77 (0.48, 1.23)	0.269	0.85 (0.50, 1.46)	0.568	1.23 (0.72, 2.11)	0.457	1.18 (0.66, 2.10)	0.580	1.50 (0.94, 2.39)	0.091	
Services (Model 3)											Model 3
Median age	**1.06 (1.01, 1.11)**	**0.029**	**1.07 (1.01, 1.14)**	**0.019**	**1.09 (1.02, 1.16)**	**0.008**	**1.09 (1.02, 1.17)**	**0.008**	1.00 (0.95, 1.07)	0.891	
Education	0.58 (0.25, 1.34)	0.200	0.54 (0.21, 1.39)	0.204	1.40 (0.46, 4.30)	0.556	0.68 (0.24, 1.98)	0.484	0.96 (0.42, 2.22)	0.924	
Health	0.86 (0.39, 1.92)	0.720	1.15 (0.48, 2.78)	0.753	0.67 (0.23, 1.99)	0.472	1.14 (0.39, 3.32)	0.815	1.18 (0.54, 2.59)	0.685	
Services	1.26 (0.77, 2.08)	0.358	1.34 (0.77, 2.32)	0.300	1.39 (0.84, 2.27)	0.196	1.05 (0.64, 1.74)	0.837	1.25 (0.78, 2.00)	0.361	
Commercial (Model 4)											Model 4
Median age	**1.07 (1.02, 1.12)**	**0.010**	**1.08 (1.02, 1.15)**	**0.008**	**1.09 (1.02, 1.16)**	**0.011**	**1.09 (1.02, 1.17)**	**0.007**	1.00 (0.95, 1.06)	0.936	
Industry	0.73 (0.38, 1.42)	0.356	0.86 (0.41, 1.81)	0.697	0.76 (0.40, 1.45)	0.408	0.83 (0.40, 1.72)	0.615	0.85 (0.48, 1.51)	0.590	
Retail	1.02 (0.53, 1.95)	0.957	1.07 (0.52, 2.22)	0.852	1.15 (0.62, 2.14)	0.657	1.30 (0.64, 2.61)	0.465	1.29 (0.74, 2.26)	0.371	
Sports and Recreation (Model 5)											Model 5
Median age	**1.06 (1.01, 1.12)**	**0.012**	**1.08 (1.01, 1.15)**	**0.017**	**1.08 (1.01, 1.15)**	**0.026**	**1.13 (1.05, 1.22)**	**0.001**	1.00 (0.95, 1.06)	0.952	
Arena	**1.95 (1.21, 3.15)**	**0.006**	1.33 (0.76, 2.32)	0.325	0.91 (0.53, 1.55)	0.723	**2.03 (1.05, 3.91)**	**0.034**	1.26 (0.80, 2.01)	0.320	
Oval	0.62 (0.36, 1.06)	0.079	0.79 (0.42, 1.48)	0.456	**2.05 (1.11, 3.79)**	**0.021**	1.25 (0.62, 2.53)	0.527	1.33 (0.80, 2.18)	0.269	
Sports and recreation	0.83 (0.51, 1.36)	0.461	0.89 (0.51, 1.56)	0.691	0.84 (0.50, 1.39)	0.487	0.69 (0.41, 1.16)	0.161	1.05 (0.66, 1.66)	0.847	
Disused (Model 6)											Model 6
Median age	**1.06 (1.01, 1.12)**	**0.015**	**1.08 (1.02, 1.14)**	**0.012**	**1.09 (1.02, 1.16)**	**0.008**	**1.10 (1.03, 1.17)**	**0.005**	1.01 (0.95, 1.06)	0.851	
Disused	**0.65 (0.42, 1.00)**	**0.048**	0.77 (0.47, 1.26)	0.303	1.20 (0.75, 1.93)	0.437	1.16 (0.70, 1.91)	0.572	1.27 (0.83, 1.93)	0.266	
Geographic Isolation (Model 7)	(*n* = 83)		(*n* = 83)		(*n* = 66)		(*n* = 58)		(*n* = 77)		Model 7
Median age	**1.07 (1.01, 1.12)**	**0.014**	**1.08 (1.02, 1.15)**	**0.014**	**1.11 (1.03, 1.19)**	**0.004**	**1.12 (1.03, 1.21)**	**0.007**	1.01 (0.94, 1.07)	0.851	
AGIL communities (300 km) *	**4.68 (1.55, 14.16)**	**0.006**	**6.10 (1.12, 33.11)**	**0.036**	1.44 (0.33, 6.19)	0.625	0.72 (0.10, 5.23)	0.745	1.75 (0.69, 4.41)	0.237	

Note—boldface indicates statistical significance at *p* = 0.05. AGIL—Australian Government Indigenous Programs and Policy Location, CMD—Cardiometabolic disease, CI—confidence intervals, ED—Emergency Department, IRR—Incidence Rate Ratio, *—mainland communities only.

## Data Availability

Not applicable.

## Supplementary Materials

The following supporting information can be downloaded at: https://www.mdpi.com/article/10.3390/ijerph19159435/s1, Table S1: EnRICH health outcomes documentation.
