# Supplementary material for: Built Environment Features and Cardiometabolic Mortality and Morbidity in Remote Indigenous Communities in the Northern Territory, Australia
Source: Int J Environ Res Public Health. 2022 Aug 1;19(15):9435. doi: 10.3390/ijerph19159435 (PMC9368214; doi:10.3390/ijerph19159435)
Supplement: Supplementary file 1 [file ijerph-19-09435-s001.zip › ijerph-1798825-supplementary.pdf]

Supplementary Materials: Built environment features and cardiometabolic mortality and morbidity in remote Indigenous communities in the Northern Territory, Australia

Authors: Amal Chakraborty*, Margaret Cargo, Victor Maduabuchi Oguoma, Neil T. Coffee, Alwin Chong, and Mark Daniel

Table S1. EnRICH health outcomes documentation.

**Definition of health outcome measures**
A. Individual de-identified comprehensive cardiometabolic disease and risk profile data from the Northern Territory (NT) Department of Health for the period 1 January 2010 to 31 December 2015, including:<br>1) Inpatient Activity (diagnosis and procedures at a hospital)<br>2) Emergency Department Data Collection (presentation at a hospital emergency department)<br>3) Primary Health Care Collection (diagnosis/clinical assessment at primary health care centre)<br><br>B. De-identified mortality data from the Births, Deaths, and Marriages Office (NT Department of the Attorney-General and Justice) for the period 1 January 2010 to 31 December 2015.<br>1) all-cause mortality, and<br>2) and cardiovascular-specific
For ***A1 Inpatient Activity*** (*diagnosis and procedures at a hospital*), and ***A2 Emergency Department Data Collection*** (*presentation at a hospital emergency department*):<br>Study population = all patients with ICD-10 diagnosis codes<br>E11 Type 2 diabetes mellitus<br>E16 Other disorders of pancreatic internal secretion<br>E66 Obesity<br>E78 Disorders of lipoprotein metabolism and other lipidaemias E88 Other metabolic disorders<br>I10 Essential (primary) hypertension<br>I11 Hypertensive heart disease<br>I12 Hypertensive renal disease<br>I13 Hypertensive heart and renal disease<br>I15 Secondary hypertension<br>I20 Angina pectoris<br>I21 Acute myocardial infarction<br>I22 Subsequent myocardial infarction<br>I23 Certain current complications following acute myocardial infarction<br>I24 Other acute ischaemic heart diseases<br>I25 Chronic ischaemic heart disease<br>I26 Pulmonary embolism<br>I27 Other pulmonary heart diseases<br>I28 Other diseases of pulmonary vessels<br>I60 Subarachnoid haemorrhage<br>I61 Intracerebral haemorrhage<br>I62 Other nontraumatic intracranial haemorrhage<br>I63 Cerebral infarction<br>I64 Stroke, not specified as haemorrhage or infarction<br>I65 Occlusion and stenosis of precerebral arteries, not resulting in cerebral infarction<br>I66 Occlusion and stenosis of cerebral arteries, not resulting in cerebral infarction

Definition of health outcome measures
I67 Other cerebrovascular diseases<br>I68 Cerebrovascular disorders in diseases classified elsewhere<br>I69 Sequelae of cerebrovascular disease<br>I70 Atherosclerosis<br>I71 Aortic aneurysm and dissection<br>I72 Other aneurysm and dissection<br>I74 Arterial embolism and thrombosis<br>I77 Other disorders of arteries and arterioles<br>I79 Disorders of arteries, arterioles and capillaries in diseases classified elsewhere<br>N00 Acute nephritic syndrome<br>N01 Rapidly progressive nephritic syndrome<br>N02 Recurrent and persistent haematuria<br>N03 Chronic nephritic syndrome<br>N04 Nephrotic syndrome<br>N17 Acute renal failure<br>N18 Chronic renal failure<br>N19 Unspecified kidney failure<br><br>**Inpatient Activity** coded for patient unique ID, DOB, gender, Indigenous status, Community name (suburb or locality) as text (usual or most recent residence), discharge date, all discharge diagnoses – ICD-coded, all discharge diagnoses – text, ICD version number.<br><br>**Emergency Department Data Collection** coded for patient unique ID, DOB, gender, Indigenous status, Community name (suburb or locality) as text (usual or most recent residence), presentation diagnosis date, presentation diagnosis – ICD-coded, ICD version number.
For ***A3 Primary Health Care Collection*** (*diagnosis/clinical assessment at primary health care centre*)<br>Study population = ICPC Diagnosis code relating to cardiometabolic and behavioural risk factors<br>ICPC-2 Rubric including ICPC-2 PLUS codes where indicated (ICPC-2 Rubric label)<br>T89 (Diabetes; insulin-dependent)<br>T90 (Diabetes; non-insulin-dependent)<br>W85 (Gestational diabetes)<br>K85 (Elevated blood pressure (without hypertension)<br>K86 (Hypertension; uncomplicated)<br>K87 including ICPC-2 PLUS codes W81002-W81003 (Hypertension; complicated)<br>K74 (Ischaemic heart disease with angina)<br>K76 (Ischaemic heart disease without angina)<br>A34005 (Test; C reactive protein)<br>D34005 (Test; aspartate aminotransferase)<br>K34003 (Test; cardiac enzymes)<br>K34004 (Test; creatine kinase)<br>T34010 (Test; HbA1c)<br>T34017 (Test; fructosamine)<br>T34022 (Test; HBA1)<br>T34005 (Test; glucose)<br>T34009 (Test; glucose tolerance)<br>T34023 (Test; glucose (fasting/random))

Definition of health outcome measures
T34025 (Test; glucose; fasting)<br>T34026 (Test; glucose; random)<br>B34026 (Test; fibrinogen)<br>K86 (Hypertension; uncomplicated)<br>K87 (Hypertension; complicated)<br>K74 (Ischaemic heart disease with angina)<br>K75 (Acute myocardial infarction)<br>K76 (Ischaemic heart disease without angina)<br>T93 including ICPC-2 PLUS codes T99075 (Lipid disorder)<br>T82 (Obesity)<br>T83 (Overweight)<br>K84017 (Cardiomyopathy; primary)<br>K84034 (Cardiomyopathy; secondary)<br>K84035 (Cardiomyopathy; congestive)<br>K84036 (Cardiomyopathy; restrictive)<br>K84037 (Cardiomyopathy; hypertrophic)<br>K84041 (Cardiomyopathy)<br>K74 (Ischaemic heart disease with angina)<br>K75 (Acute myocardial infarction)<br>K76 (Ischaemic heart disease without angina)<br>K77 (Heart failure)<br>K78 (Atrial fibrillation/flutter)<br>K79 (Paroxysmal tachycardia)<br>K80 (Cardiac arrhythmia NOS)<br>K83 (Heart valve disease NOS)<br>K84 including ICPC-2 PLUS codes K03001 - K99032 (Heart disease, other)<br>K06 (Prominent; veins)<br>K22 (Risk factor; cardiovascular disease)<br>K85 (Elevated blood pressure)<br>K86 (Hypertension, uncomplicated)<br>K87 (Hypertension, complicated)<br>K88 (Postural hypotension)<br>K92 (Atherosclerosis/peripheral vascular disease)<br>K93 (Pulmonary embolism)<br>K94 (Phlebitis/thrombophlebitis)<br>K89 (Transcient cerebral ischaemia)<br>K90 (Stroke/cerebrovascular accident)<br>K91 (Cerebrovascular disease)<br>W85 (Gestational diabetes)<br><br>**Primary Health Care Collection** coded for patient unique ID, current age, diagnosis date, Indigenous status, usual clinic name (attributed to suburb/locality and aggregated to form community-level outcomes), suburb/locality associated with clinic, residency status (resident, visitor, or tourist), ICPC Diagnosis code.

Definition of health outcome measures
**For B Territory-wide Mortality data**<br><br>1) all-cause mortality (see: http://apps.who.int/classifications/icd10/browse/2010/en)<br>2) cardiovascular-specific mortality, including:<br>• ischaemic heart disease (ICD-10 codes I20–I25)<br>• cerebrovascular disease (ICD-10 codes I60–I69), and<br>• diseases of the arteries, arterioles and capillaries (ICD-10 codes I70–I78)
